# CohortFinder: an open-source tool for data-driven partitioning of digital pathology and imaging cohorts to yield robust machine-learning models

**DOI:** 10.1038/s44303-024-00018-2

**Published:** 2024-07-01

**Authors:** Fan Fan, Georgia Martinez, Thomas DeSilvio, John Shin, Yijiang Chen, Jackson Jacobs, Bangchen Wang, Takaya Ozeki, Maxime W. Lafarge, Viktor H. Koelzer, Laura Barisoni, Anant Madabhushi, Satish E. Viswanath, Andrew Janowczyk

**Affiliations:** 1 Emory University and Georgia Institute of Technology, Department of Biomedical Engineering, Atlanta, GA USA; 2 Case Western Reserve University, Department of Biomedical Engineering, Cleveland, OH USA; 3 Duke University, Department of Pathology, Division of AI & Computational Pathology, Durham, NC USA; 4 University of Michigan, Department of Internal Medicine, Division of Nephrology, Ann Arbor, MI USA; 5 University Hospital of Zurich, University of Zurich, Department of Pathology and Molecular Pathology, Zurich, Switzerland; 6 Duke University, Department of Medicine, Division of Nephrology, Durham, NC USA; 7 Atlanta Veterans Administration Medical Center, Atlanta, GA USA; 8 University Hospital of Geneva, Department of Oncology, Division of Precision Oncology, Geneva, Switzerland; 9 University Hospital of Geneva, Department of Clinical Pathology, Division of Clinical Pathology, Geneva, Switzerland

**Keywords:** Image processing, Biomedical engineering

## Abstract

Batch effects (BEs) refer to systematic technical differences in data collection unrelated to biological variations whose noise is shown to negatively impact machine learning (ML) model generalizability. Here we release CohortFinder (http://cohortfinder.com), an open-source tool aimed at mitigating BEs via data-driven cohort partitioning. We demonstrate CohortFinder improves ML model performance in downstream digital pathology and medical image processing tasks. CohortFinder is freely available for download at cohortfinder.com.

The increased availability of digital pathology (DP) whole slide images (WSI) and radiographic imaging datasets has propelled the development of both machine and deep learning algorithms to aid in disease diagnosis, patient prognosis, and predicting therapy response^1^. These algorithms work by identifying patterns in digital data that are associated with clinical outcomes of interest. While large-scale data analysis was previously limited by storage, processing, and computational constraints, modern-day development and testing of these models increasingly involves the collection of large cohorts over both physical (e.g., institutions) and temporal (e.g., time points) spaces^1^. However, differences in non-biological preanalytical processes at these various spatiotemporal points likely impart undesirable batch effects (BE) in the final digital data. For example, BEs in DP images generated in the same manner from the same tissue type yield significant visual differences which may impact data interpretation (see Fig. 1A).

**Fig. 1:**
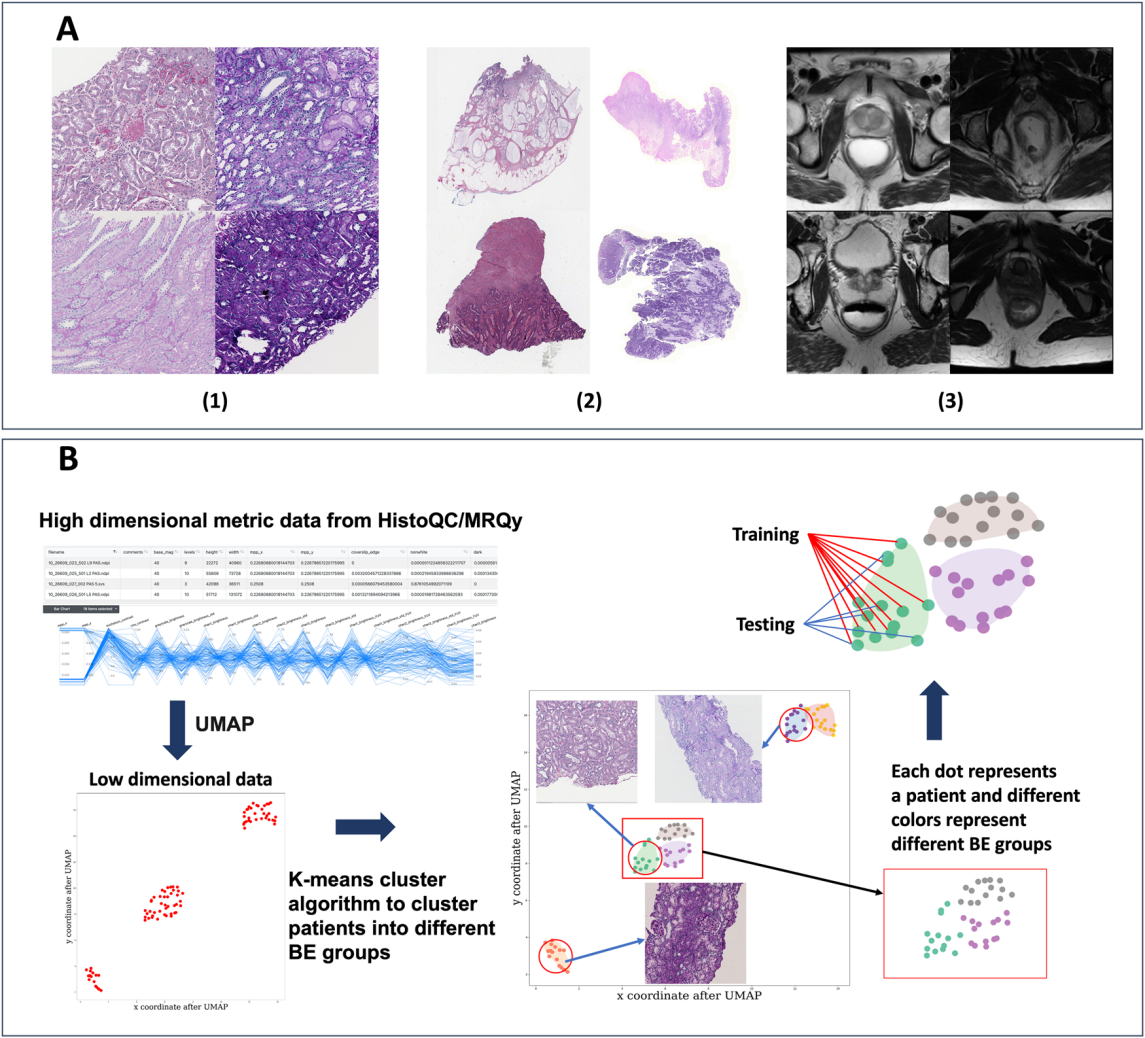
Batch effect examples and workflow for CohortFinder in digital pathology and radiology domains. **A** Examples of the batch effects with (1) four ROIs from the tubule segmentation task, (2) four WSI thumbnails from the colon adenocarcinoma detection task, and (3) four images sections from four different patients from the rectal cancer segmentation task. As can be seen, the DP images show notable differences in white balance, brightness, and contrast demonstrating clear BEs. Similarly, the MRI imaging data also shows significant differences in foreground contrast. **B** The basic workflow for CohortFinder. First, UMAP is used to project high-dimensional quality control metric values into a two-dimensional space. Second, k-means clustering takes place in this two-dimensional space to identify BE-groups using approximately k target clusters. Finally, patients in each BE group are assigned to a training/testing set based on the user-given ratio while sampling from each BE group.

In DP, these BEs tend to originate from, but are not limited to, differences in physical processes for data generation (tissue processing, storage, glass slide preparation) as well as digitization processes (scanners, color profile management, compression approaches)^1–6^. In MR imaging cohorts, these BEs may result from MRI acquisition protocols, patient preparation differences, or imaging artifacts such as noise, motion, inhomogeneity, ringing, or aliasing^7,8^. Regardless of the modality, BEs have been shown to not only severely hamper both the peak performance and robustness (i.e., degree of performance change when examining new unseen data) of machine learning (ML) models but can also cause spurious discoveries when associated with outcome variables of interest^5,9,10^. For example, ML has been shown to be able to detect and thus be influenced by BEs associated with the site of origin^11,12^, potentially leading to biased accuracy in the prediction of survival, genomic mutations, or tumor stage.

Given the detrimental impact of BEs, there have been several approaches developed for ameliorating them. For example, (a) ComBat^7^, (b) Generative Adversarial Networks (GAN)^3,13^, and (c) data augmentation approaches have been commonly utilized to mitigate batch effects in digital pathology and medical image data. ComBat^7^ has been used to reduce the variability of radiomic features by considering different scan parameters as separate ‘batches’ and applying a non-parametric normalization between them; which may unfortunately introduce unintended correlations leading to higher false positive rates (FPR)^14^. GAN^3,13^ have been used to generate synthetic medical images for both CT liver images^13^ and hematoxylin and eosin (H&E) pathology images^3^; however, there is a risk of GANs “hallucinating”, i.e., generating unrealistic, or untrustworthy images that are not representative of the appearance of real disease biology^15^. Another set of techniques for managing BE focuses on data augmentation^14,16^; a suite of techniques that involves increasing the size of training cohorts through the generation of additional synthetic samples (e.g., creating variants of an image based on permutations of brightness or contrast levels of real samples). Unfortunately, this process is subject to limitations in the distribution of real samples, which may inadvertently exacerbate the impact of BEs within the cohort (see Supplementary Figure 1). BEs often impact machine learning (ML) models during the ‘data partitioning’ phase, which is defined as the ML best practice of dividing a cohort into training and testing sets. The training set is employed to create the model, while the testing set is used to determine the model’s generalizability performance on previously unseen data not employed during training.

The most common way to partition cohorts is to randomly assign patients to training and testing sets, which we term the Average Case (AC) (see Supplementary Fig. 1a). The AC strategy, however, has the potential to result in *unreasonably* sub-optimal cohort partitions by sheer chance. For example, in a Worst Case (WC), images demonstrating similar BEs may be mutually and exclusively assigned to the same training/testing split, resulting in the training data sharing minimal or no visual similarity with the testing set (see Supplementary Fig. 2b for the WC data partitioning results). Such a cohort partition is likely to result in maximally exposing the ML models to the deleterious effects of BEs, and thus yielding significantly inferior performance of the resulting ML model on the testing set. Notably, this WC represents the end point of a continuum of potential real-world sub-optimal ACs, wherein models are exposed to only a subset of the true range of BE variability in the data. It then stands to reason that there is likely a Best Case (BC) (see Supplementary Fig. 2c) on the opposite end of the continuum which maximally balances BEs to yield more representative data partitions and in turn result in more generalizable ML models. Our goal, therefore, is to develop an algorithm that systematically mitigates BEs during the data partitioning phase to consistently identify the BC partitions.

Toward addressing BEs in biomedical imaging and digital pathology data, we have developed and released CohortFinder, an open-source data-driven partitioning tool for specifically determining BC cohort partitions for training and testing ML models (https://github.com/choosehappy/CohortFinder). CohortFinder ingests quality control (QC) metrics (e.g., via HistoQC/MRQy^8,11^, two open-source QC tools for digital pathology and medical image data), and at the patient level, performs unsupervised clustering to determine BE groups which are strikingly homogenous in presentation (see Supplementary Fig. 2c). By iteratively partitioning these BE groups at a user-defined ratio into training and testing sets, CohortFinder yields highly representative and diverse partitions, which balance BEs, even in cases of minority BE groups. CohortFinder also provides the ability, when given relevant spatiotemporal labels (e.g., site origin, date of scan) or downstream outcome labels (e.g., good/poor prognosis), to statistically test for BEs and provide an associated report. CF provides a useful set of visual and quantitative outputs for BE quantification and inspection (see Supplementary Section S3, Supplementary Figs. 3, 4). To evaluate the ability of CohortFinder to yield BC data partitions, three different deep-learning use cases in DP and radiographic imaging are evaluated here: (a) tubule segmentation on kidney WSIs, (b) adenocarcinoma detection on colon WSIs, and (c) rectal cancer segmentation on MR images (see Supplementary Table 1).

For quantitative comparisons, five commonly used evaluation measures^17^ (Precision, Recall, Accuracy, IoU, and F1-score), were calculated to compare the performance of BC, AC, and WC partitioning via internal cross-validation as well as on external testing data (i.e., 1 patient from each different site or scanner) for all three use-cases separately. In Supplementary Tables 3, 4, the overall performance (average and standard deviation) and fold-specific values for each evaluation measure are reported, respectively. From the tables, WC partitioning demonstrates the worst quantitative performance in all evaluation measures compared to AC and BC partitioning, across all use cases. For example, for the colon adenocarcinoma classification use case, BC demonstrates an average F1-score improvement of 0.23 compared to WC (BC: 0.87 vs WC: 0.64) and 0.06 compared to AC (0.81) in the external testing dataset. Further, BC also results in a relatively lower standard deviation than AC for most evaluation measures (for example, BC: 0.11 vs AC: 0.21 in terms of F1 score), suggesting that CohortFinder can aid in producing more robust ML models exhibiting less variance. Similarly, for the tubule segmentation use case, BC achieves an average F1-score improvement of 0.02 compared to WC and 0.01 compared to the AC (BC:0.95 vs AC:0.94 vs WC:0.93). For the rectal cancer segmentation use case, BC outperforms WC and AC with an average increase in F1-score of 0.06 and 0.05 respectively (BC’s F1-score of 0.68 versus AC’s 0.63 and WC’s 0.62). From the violin plots (Fig. 2), the F1 scores of WCs are more dispersed as compared to the AC and BC. In most cases, while the distribution of BC is often more compact than AC, occasionally the distribution between AC and BC is similar. This observation supports the notion that data partitions generated via random sampling (i.e., AC) exist on a spectrum of BE mitigation, with some providing better or worse accounting. This spectrum also illustrates that a user employing random sampling has no way of knowing where their partitions lie on the BE mitigation spectrum. As a result, they may in fact be utilizing a WC partitioning of their data by pure chance. By contrast, CohortFinder provides users with the assurance that they have an idealized partitioning that optimally accounts for BEs in a given cohort. Figure 2 further depicts qualitative comparisons of ML model results between partitioning scenarios (WC/AC/BC) for all three use cases. For the tubule segmentation & colon adenocarcinoma classification tasks, there are fewer FPs and FNs in the BC results than those in AC and WC. For the rectal tumor segmentation use case, BC best predicts the tumor contour compared to AC and WC.

**Fig. 2:**
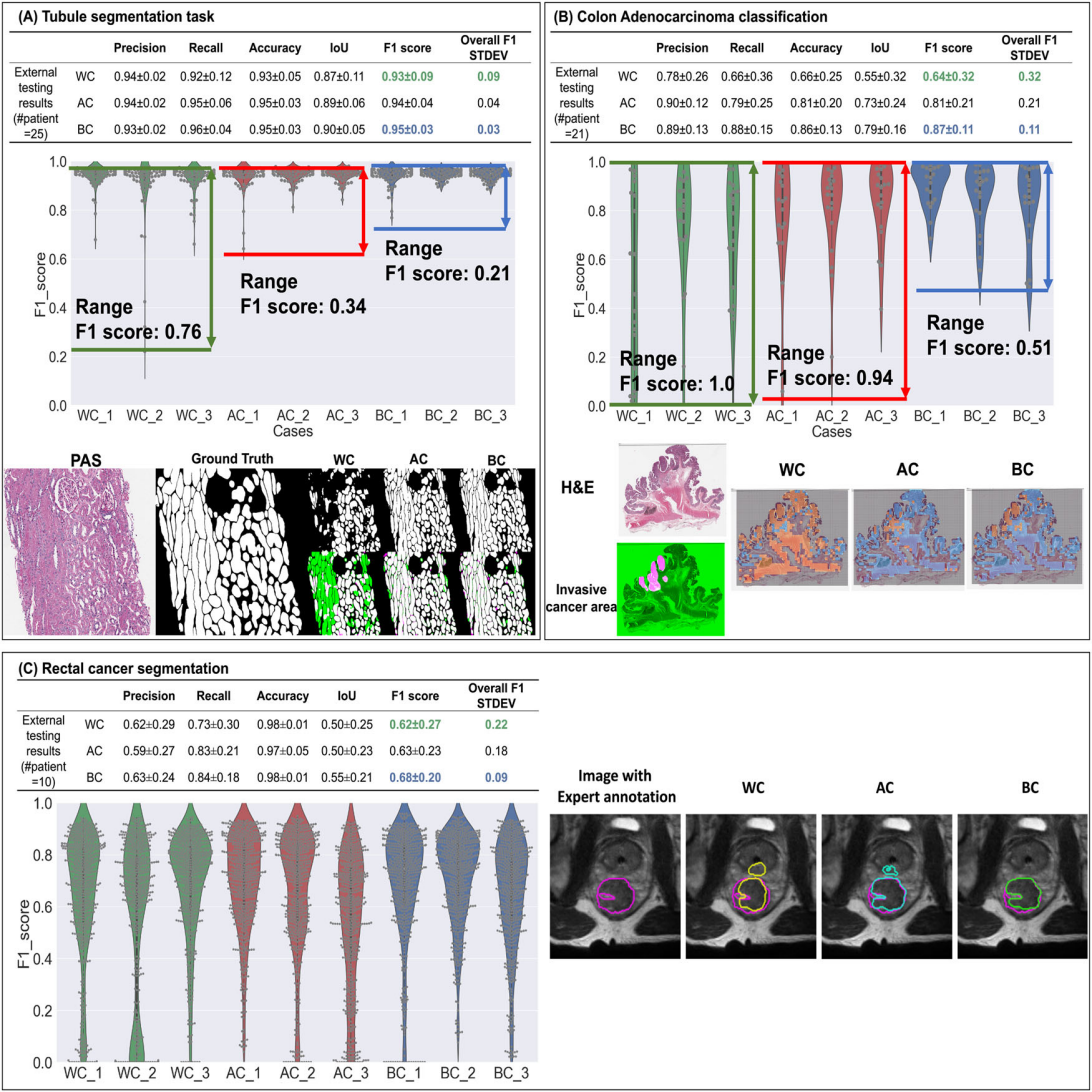
Quantitative and qualitative results for all three use cases. For each use case, we have (1) The overall performance on external testing datasets. (2) F1 score performance for 9 different models on the external testing datasets, where the gray dots in each violin plot indicate individual performance for a single image. (3) Qualitative results. In tubule segmentation task (**A**), the first column is a cropped PAS-stained image, the second column is the tubule segmentation ground truth (GT), and the remaining images are the results of WC, AC, and BC. In each scenario, the top row is the DL model result, while the bottom row corresponds to the overlay image between DL output images and the GT, where green parts represent the false negative (FN) area, and the fuchsia parts represent the false positive (FP) area. WC has more FN and FP areas compared to AC & BC. Compared to AC, BC has fewer FN and FP areas. For colon cancer classification task (**B**), the images in the first column are the H&E thumbnails and cancer annotation (the tumor area in fuchsia, non-tumor in green). The remaining three images are the heatmaps for the WC/AC/BC, where the orange area represents the predicted cancer area, blue represents the predicted no-cancer area, and the gray area represents the non-informative area (background/non-tissue area). From the heatmaps, WC over-predicts the tumor regions, AC under-predicts the tumor region, while BC yields the best overlap between tumor area and ground truth. For rectal cancer segmentation task (**C**), the first column is the image with expert annotation ground truth in fuchsia, which is also shown as a fuchsia contour in the remaining three columns. The 2D U-net segmentation results for WC (yellow), AC (cyan), and BC (green) show that WC and AC overpredict the tumor region while BC marginally underpredicts. In all three tasks, violin plots of F1 scores show a decreasing trend from BC to AC to WC. AC is also seen to have a larger F1 score range, lower average F1 value, and a higher standard deviation than BC; suggesting AC performance is less robust than BC.

Together, these results suggest that CohortFinder provides a systematic partitioning strategy that yields ML models with improved performance and generalizability by identifying representative samples covering the range of batch-effect variability available. Conversely, conventional data augmentation methods focus on simulating patterns derived from these representative samples. Therefore, we recommend users employ CohortFinder in conjunction with data augmentation (see Supplementary Fig. 1) to gain the unique benefits of the two processes. Users can also incorporate CohortFinder into their existing pipelines seamlessly; for instance, Ooijen et al.^18^. and Triguero et al.^19^. detail a general AI pipeline where CohortFinder could be integrated into the training phase, and could help transform ‘big data’ into ‘smart data’ for efficient data mining. Given the low computational burden associated with its usage (i.e.,1–2 min on a consumer-grade laptop), we believe CohortFinder will serve as a valuable tool to help avoid sub-optimal WC-like cohort partitions, by replacing the typically used approach of random sampling when creating data partitions.

To summarize, we have presented and released an open-source data-partitioning tool termed CohortFinder. CohortFinder works by identifying potential batch-effect groups and ensuring their proportional representation when partitioning a cohort into training and testing sets, yielding demonstrably more reliable downstream ML models in batch-effect-laden datasets. CohortFinder’s BE-groups can also facilitate rapid identification of representative samples to bootstrap downstream workflows, such as annotation. Importantly, CohortFinder ingests input metrics in a common TSV format, produced by open-source quality control tools (HistoQC/MRQy^8,11^). This suggests that as our knowledge of batch effects and quality control improves, and more sophisticated metrics are developed, CohortFinder will be organically capable of leveraging them for further improving downstream ML models. The source code for CohortFinder is freely available for use, modification, and contribution at cohortfinder.com.

## Methods

### Data partitioning based on BE groups

CF proceeds by identifying multiple BE “groups”, i.e., sets of images with similar presentation metrics as calculated by HistoQC^11^/MRQy^8^ (two open-source quality control tools for pathology and radiology, respectively). These BE groups are then iteratively randomly divided into subsets at user-specified ratios (the ratio of the testing data and all the data). As a result, the training and testing sets have balanced representations of BE variability to help ensure diversity for ML models.

A key component in consistently generating BC data partitions is the ability to detect BEs a priori. While this can be approximated using either available metadata (e.g., site or scanner labels) or by visual assessment, the labor of labeling involved quickly becomes intractable and non-reproducible for large datasets. This also does not leverage the most critical source of information readily available, the presentation of the images themselves. Importantly, previous work has demonstrated that computationally derived quality control (QC) metrics can be repurposed to detect BEs^8–12^.

### HistoQC/MRQy functionality

CohortFinder utilizes the output from either HistoQC or MRQy, open-source tools designed to aid in QC of digital pathology and imaging modalities (e.g., MRI, CT, PET), respectively^8,11^. These tools allow for large-scale high-throughput extraction of deterministic image quality measures. In both modalities, images are sequentially fed into the pipeline where each module (a) captures basic metadata (e.g., the base magnification, the microns per pixel, repetition time, echo time, number of slices per volume), (b) quantifies visual characteristic metrics (e.g., brightness, contrast, mean of the foreground, contrast per pixel), and (c) locates artifacts (e.g., air bubbles, pen markings, noise, and inhomogeneity). The resulting metrics form the input for CohortFinder and are used to identify BE groups.

### CohortFinder functionality

Figure 1B illustrates the basic CohortFinder workflow, proceeds as follows:

- CohortFinder loads the extracted QC measures.
- The QC measures are considered a high-dimensional vector and projected into 2 dimensions via uniform manifold approximation and projection (UMAP)^20^ for visualization as a 2-dimensional embedded plot. UMAP works by modeling the data as a fuzzy topological manifold, which allows it to capture complex relationships between data points. It then optimizes a low-dimensional projection of the data, aiming to preserve both the local and global structure of the manifold. UMAP was chosen for its favorable properties over other dimensionality reduction techniques (such as t-SNE):

- *Embedding new data:* UMAP^21^ can embed new data into an existing manifold and avoid recalculating the entire model. Other methods, such as t-SNE^22^, will need a full re-computation of the entire t-SNE process for new data and thus have the possibility of resulting in vastly different embeddings. Though principal component analysis (PCA) can also be used to transform new data with higher speed^23,24^, because PCA tends to ignore variation along directions other than the one with maximum variation, it can potentially obscure finer-scale patterns in the raw data^25^. By contrast, UMAP embeddings better capture subtle features of data and can perform better in visualization and downstream clustering tasks^23–25^.
- *Computational efficiency:* UMAP outperforms t-SNE in computational speed^26^, especially with large datasets due to its graph-based approach.
- *Sensitivity to hyperparameters:* t-SNE is more sensitive to the hyperparameter settings, such as perplexity^26^, rendering it less generalizable than UMAP.
- *Preservation of global data structure:* UMAP tends to preserve both the global and local structure of the raw data topology, aiding in a deeper understanding of the overall relationships and structures within the data.

(c) *K*-means^27^ clustering takes place in this 2-dimensional UMAP space to identify *k* target clusters where each cluster is considered to represent a BE group. Replicated clustering was used here to mitigate the impact of k-means’ randomness and improve the stability of the clustering algorithm. K-means^27^ was utilized here due to its high computational efficiency and easy implementation. Furthermore, the results produced by K-means are intuitive, as each data point is assigned to the nearest cluster center. In the future, it could be valuable to consider experimenting with other clustering algorithms within a more comprehensive ablation study.
(d) For each BE group, images are randomly assigned into training and testing according to a user-specified ratio.

CF produces four outputs: (1) UMAP plots (shown in Supplementary Figure 3) indicate colored BE-group distribution results in 2-dimensional UMAP space based on QC measures (Supplementary Fig. 3a). (2) patient assignment results for training and testing (Supplementary Fig. 3b) where “v” indicates a patient to be placed in the training set versus “o” to indicate the testing set, (2) a contact sheet type image (shown in Supplementary Figure 4) with representative images from each BE-group, (3) a general log containing information for the user as well as potential errors, and (4) a comma-separated value file that contains (a) the metrics used to perform the BE-group detection, (b) the resulting UMAP coordinates, (c) the determined BE-group index number, (d) the label assigned to the particular image (e.g., training vs testing), and (e) 3 different clustering metrics to help quantify batch-effect severity (see Supplementary Section S3).

### Batch-effect testing module of CohortFinder

If the user also provides labels of interest (e.g., the site information where the images are collected from, or clinical variable of interest), CohortFinder runs a permutation test for the presence of BEs. Similar to previous study^12^, this approach utilizes a random forest (RF) machine learning model based on HistoQC/MRQy metrics to predict the origin of images (e.g., it will determine whether it’s possible to classify images from each origination site based on quality metrics) and to rank these metrics by their importance as predictors of factors that drive BEs. The performance of the RF model is compared against an RF model trained using randomized labels. The null hypothesis for this test suggests that if the prediction results using the specific image labels (e.g., origination sites) are not significantly better than those obtained with random labels, there is an absence of BEs associated with HistoQC/MRQy metrics.

### Experimental design

To evaluate the ability of CohortFinder to yield optimal data partitions, three different deep-learning use cases in DP and medical imaging areas were selected: (a) tubule segmentation on kidney WSIs, (b) adenocarcinoma detection on colon WSIs, and (c) rectal cancer segmentation on MR images (see Supplementary Table 1 for detailed description for the 3 use cases). For each use case, 1 patient from each site/scanner was randomly selected to be included in a benchmark external testing set while the remaining patients were used for developing training and testing partitions. For the latter, three scenarios were explored:

- *Best Case (BC):* Patients were segregated via CohortFinder into BE groups, following which equitable BE distribution of samples was ensured in all partitions. The number of clusters (*k*) in CohortFinder was set to the number of patients divided by 3, to allow for 3-fold cross-validation where 1 patient from each 3-patient cluster is assigned to 1 of the 3 cross-validation folds (BC_1, BC_2, and BC_3). For example, in the tubule use case which had 91 patients, the CohortFinder-cluster parameter was set to 31.
- *Average Case (AC):* As per typical ML practices, samples were randomly split into 3 average-case folds (AC_1, AC_2, and AC_3) without considering BEs.
- *Worst Case (WC):* Patients were segregated to intentionally maximize the BE differences, as defined by HistoQC/MRQy metrics, between each worst-case group (WC_1, WC_2, and WC_3). To do so, patients were clustered into 3 BE groups, yielding groups that are notably different in presentation. Similar to how we determined BC, CohortFinder was used here to cluster the patients into 3 BE groups, where each BE group had a similar number of patients. This allowed for 3-fold cross-validation where the patients in one BE group were assigned to 1 of the 3 cross-validation folds (WC_1, WC_2, and WC_3). For example, in the tubule use case which had 91 patients, each BE group was comprised of 31/30/30 patients, respectively.

During the experimental evaluation, internal patient-level cross-validation took place with each of the folds serving as the training set, and the derived model was subsequently evaluated on the remaining folds as an “internal” testing set. For example, AC_1 was used to train an ML model which was then evaluated on AC_2 and AC_3. This internal testing process was conducted to gain a robust estimate of ML model performance within the training set. Patient-level distribution ensured that images from the same patient only appeared in a specific fold and were not distributed across folds. Each trained ML model was also tested on the external testing cohort, allowing for a fair cross-fold comparison.

### Evaluation metrics

Five metrics were used to evaluate model performance: precision, recall, accuracy, IOU, and F1 score, based on their wide usage in ML model-based segmentation and classification tasks^17^. Before calculating the metric value, true positive (TP), true negative (TN), false positive (FP), and false negative (FN) predictions were calculated at a pixel level (for two segmentation use cases) and patch level (for the classification use case). Each metric was subsequently measured following the formulas in Supplementary Table 2).

### Network configuration and training

U-net^28,29^ was used for the segmentation tasks and Dense-Net^30^ was used for the classification task, selected based on their popularity for these tasks. Both architectures were implemented in PyTorch with the following configuration details:

1. Tubule segmentation: (a) depth of the U-Net: 5 blocks, number of filters in the filter layer: 4, (b) patch size: 512 × 512, number of training batches for each epoch: 6, (c) number of training epochs: 50, the model with the lowest validation loss was used to do the testing, (d) optimization algorithm: Adam and (e) data augmentation: vertical & horizontal flips and rotation were used during the network training process.
2. Colon adenocarcinoma classification: (a) Dense-Net architecture: growth rate is 32, drop rate is 0, initial feature number is 64, batch norm size is 2, (b) patch size: 224, number of training batches for each epoch: 64, (c) number of training epochs: 50, the model with lowest validation loss was used for the testing, (d) optimization algorithm: Adam and (e) data augmentation: vertical & horizontal flips and rotation were used during the network training process.
3. Rectal cancer segmentation: (a) depth of U-Net: 5 blocks, number of filters: 30, (b) size of cropped input images: 128 by 128, batch size: 16, (c) 50 epochs specified but with early stopping implemented based on the dice similarity coefficient loss function with a patience of 4 epochs, model with lowest validation loss was used for testing (d) optimization algorithm: Adam and (e) data augmentation: vertical & horizontal flips and rotation.

## Supplementary information

Supplementary Information

## Data Availability

No datasets were generated or analysed during the current study.

## Code availability

CohortFinder is available at: https://cohortfinder.com Detailed documentation and usage instructions are included in the repository to facilitate reproducibility and further research.
