# Supplementary material for: CohortFinder: an open-source tool for data-driven partitioning of digital pathology and imaging cohorts to yield robust machine-learning models
Source: Npj Imaging. 2024 Jul 1;2:15. doi: 10.1038/s44303-024-00018-2 (PMC11216973; doi:10.1038/s44303-024-00018-2)
Supplement: Supplementary file 1 — Supplementary Information [file 44303_2024_18_MOESM1_ESM.pdf]

Supplementary materials

S1. Datasets

Tubule segmentation on kidney WSIs

Cohort description

Kidney WSIs were obtained from the Nephrotic Syndrome Study Network (NEPTUNE) digital pathology repository. NEPTUNE is a multisite observational cohort study of children and adults with glomerular disease, enrolled at the time of a clinically indicated kidney biopsy[1-4]. The renal biopsies were processed in 38 different pathology laboratories, collected, and shipped to the NEPTUNE image coordinating center, where glass slides were centrally scanned by two scanners (Aperio Scanscope AT2, Leica Biosystems Inc., Buffalo Groove, IL, USA and Hamamatsu Nanozoomer 2.0 HT, Hamamatsu Corporation, Hamamatsu City, Japan; both with an Olympus UPlan-SApo 20X objective, with a 0.75 NA, and image doubler) and subsequently uploaded into the NEPTUNE DPR. In this use case, 116 Periodic acid–Schiff (PAS) stained WSIs from 25 sites were included. WSIs were chosen such that each patient contributed one randomly selected WSI.

Ground-truth generation

For each WSI, 3 ROIs (region of interest) from the cortical area containing renal tubules were randomly selected and manually cropped as 3000 x 3000 tiles at 40x digital magnification. A pre-trained DL model[5] was implemented on each tile to acquire an approximate tubule segmentation result. Two renal pathologists then manually evaluated all tubule segmentation results and revised them until all the tiles' results reached at least less than 5% FN/FP at the tubule level. **Supplementary Figure 5** shows one example of the tubule segmentation use case.

Colon adenocarcinoma classification on colon WSIs

Cohort description

Colon WSIs were obtained from The Cancer Genome Atlas (TCGA) Colon Adenocarcinoma (COAD) cohort, a publicly available repository (https://portal.gdc.cancer.gov/projects/TCGA-COAD). 26 WSIs were excluded during the quality control (QC) process because of low base magnification or bad quality. For this use case, a total of 352 whole slide images (WSIs) were selected, all of which contained diagnostic information indicating the presence of 'Adenocarcinoma, NOS'. WSIs were chosen such that each patient contributed one WSI, if a patient has multiple slides, the slide of the highest quality (as determined by the quality control pipeline described in previous work[1]) was chosen.

Ground-truth generation

For every WSI, a senior pathologist manually annotated representative areas of colorectal adenocarcinoma to be used in an image-based molecular classification task[6], and the training ground-truth patches were generated based on this expert annotation. For the patch sampling process, HistoQC[7] was used to generate the tissue mask image. **Supplementary Figure 6-A. (1)** illustrates this process for a tissue mask image generated by HistoQC for a specific patient (id: TCGA-F4-6805-01Z). All the potential patches (256 x 256 at 5x magnification) were generated from the WSI before being assigned a ground truth label via tessellation. Tessellation here, in the context of processing a WSI, means dividing a large image into smaller, non-overlapping, square patches. **Supplementary Figure 6-A. (2)** shows all the potential patches mapped back to the thumbnail. Patches were retained for training if (a) >90% area was intersected with the detected tissue mask, and (b) color density maximum differences for all the 3 color channels were greater than 20. Otherwise, they were labeled as 'non-informative' and excluded from the cohort. Patches were labeled as positive for cancer if >90% of the patch fell within the ground-truth annotation for cancer. Otherwise, the patch was labeled as negative for cancer. **Supplementary Figure 6-B** shows representative cancer and non-cancer patches from one WSI, which were utilized for the experimental evaluation of CohortFinder.

Rectal cancer segmentation on MRIs

Cohort description

MRI scans were acquired from 166 patients diagnosed with rectal adenocarcinoma between August 2007 and October 2015, who had been retrospectively accrued from two institutions (University Hospitals Cleveland Medical Center and Cleveland Clinic, OH, USA). Based on IRB approval, informed consent was waived as all data had been deidentified prior to analysis. These MRI scans had been acquired prior to neoadjuvant chemoradiation treatment for primary staging of the rectal tumor, via a T2-weighted turbo spin echo sequence (T2w) in the axial plane on ten unique scanners from two different manufacturers (Philips and Siemens). Despite scanner variability, imaging parameters were fairly consistent within each institution (in-plane resolution: 0.313-1.172mm, slice thickness: 3.0-8.0mm, repetition time: 2400-11800msec, echo time: 64-184msec).

Ground-truth generation

Annotations of rectal tumor extent on each T2w MRI dataset were obtained from a radiologist at each institution, who had access to clinical, pathologic, and radiology reports, as well as any additional imaging planes and sequences. Radiologists annotated the entirety of the gross tumor volume on all 2D slices between the peritoneal reflection and the top of the anal canal using 3D Slicer[8]. To minimize the effect of resolution differences within this cohort, all patient datasets and corresponding tumor annotations were resampled to a common resolution of 1.00 x 1.00 x 1.00mm. After resampling, 2D slices without tumor annotations were excluded, while the remaining 7897 2D slices were cropped to a uniform 128x128 bounding box centered on the tumor region.

S2. Results

Tubule segmentation

Quantitative result

In the external testing set (n=25 patients), the best F1 overall score results were from BC partitioning (0.95±0.03), followed by AC (0.94±0.04), and finally WC (0.93±0.09), with statistically significant differences between WC and AC ($p<0.01$) as well as between WC and BC ($p<0.01$). While no statistically significant differences were observed between AC and BC ($p=0.71$), AC resulted in a larger range of F1 scores (the violin plot in **Figure 2-A**), a lower average F1 value, and a higher standard deviation in F1 scores (the table in **Figure 2-A** shows the overall average ± standard deviation results for external testing results in terms of all the measurements for all the 3 use cases) compared to BC. This suggests less robust performance for AC compared to BC.

Qualitative result.

In **Figure 2-A** and **Supplementary Figure 7-A**, WC partitioning results in a relatively higher number of false negative (FN) areas (overlayed green regions) in comparison to AC and BC. Additionally, AC yielded a marginally higher number of false positive (FP) and FN regions (highlighted in fuchsia and green, respectively) when compared to BC.

Colon adenocarcinoma classification

Quantitative result

In the external testing dataset (n=21 patients), the F1 score is seen to be significantly higher when comparing BC and WC (0.87±0.11 vs 0.64±0.32, $p<0.01$) as well as between AC and WC (0.81±0.21 vs 0.64±0.32, $p<0.01$). Though no significant differences were found between BC and AC (0.87±0.11 vs 0.81±0.21, $p = 0.09$), the violin plots in **Figure 2-B** and **Supplementary Figure 8** suggest that BC has a more compact F1 score distribution, a higher average F1 score, as well as a lower standard deviation compared to AC.

Qualitative result.

In **Figure 2-B** and **Supplementary Figure 7-B**, classification heatmaps produced via BC partitioning exhibit the highest degree of similarity with the ground-truth mask. WC partitioning resulted in a significant underprediction of the tumor area, with a considerable number of false negative (FN) patches within normal tissue. AC partitioning yielded a slightly smaller prediction of tumor area when compared to BC.

Rectal cancer segmentation

Quantitative result

In the external testing dataset (n=10 patients), BC models resulted in the highest overall F1 score of 0.68±0.20, while the AC and WC models yielded significantly lower overall F1 scores of 0.63±0.23 ($p<0.01$ vs BC) and 0.62 ±0.20 ($p<0.01$ vs BC), respectively (**Figure 2-C** shows these measurements for the rectal cancer segmentation task). The markedly higher standard deviation in F1 scores of the WC models is illustrated in the violin plots of **Supplementary Figure 8-C**. Notably, the bottom tails of the F1 score distribution for WC models (green) are seen to be longer and wider in comparison to those of the AC (red) and BC models (blue). This suggests that tumor segmentations by WC models were more varied and shared little overlap with expert annotations, resulting in marked variations in model performance compared to AC and BC.

Qualitative result.

Figure 2-C and **Supplementary Figure 7-C** depict representative tumor segmentations obtained via WC, AC, and BC partitioning schemes compared to radiologist annotations for two different patients. BC tumor segmentations are seen to consistently overlap with expert delineations, while AC models appear to slightly over-segment the tumor region. By comparison, the WC model is seen to have a more varied performance in terms of under-segmenting or over-segmenting the tumor.

S3. Batch-effect severity evaluation (BE score)

To assist users in quantifying the severity of batch effects, we conducted a preliminary evaluation of clustering metrics to determine the segregation of detected BE-groups: (a) Silhouette coefficient, (b) Davies-Bouldin index, and (c) Calinski-Harabasz index[9]. These scores are now reported both the CohortFinder output files and in the user interface of MRQy and HistoQC**.** Initial experimental evaluation of the BE score were conducted using: (a) the entire cohort (where significant BEs may be expected to be present as it is multi-institutional), (b) site D5 (where fewer/minimal BEs are likely to be present, uni-institutional). Our preliminary results show that cohorts exhibiting the more severe BEs exhibit higher BE scores (see **Supplementary Figure 9**). In future work, we will investigate the impact of these metrics on downstream applications, such as for the selection of *k* (i.e., BE-groups).

Supplementary - Figures and Tables

Task	Modality	Dataset	Description	Evaluation metric
Tubule segmentation	Digital Pathology<br><br>PAS-stained WSI	NEPTUNE	N=116 WSIs were selected, originating from 25 different institutions. Each WSI represents one single patient. From each site, 1 patient is randomly chosen for the external testing dataset.	**Precision<br>Recall<br>Accuracy<br>IOU<br>F1 score**
Colon adenocarcinoma classification	Digital Pathology<br><br>H&E-stained WSI	TCGA-COAD	N = 352 cases were selected and diagnosed as 'Adenocarcinoma, NOS', originating from 21 different institutions. From each site, 1 patient is randomly chosen for the external testing dataset.	
Rectal Cancer segmentation	Radiographic Imaging<br><br>MR image	University Hospitals and Cleveland Clinic	N = 166 patients' MRIs were accrued from University Hospitals Cleveland Medical Center and Cleveland Clinic, originating from 10 different MRI scanner machines.10 patients (1 per MRI scanner) were chosen for the external testing dataset.	

Supplementary Table 1. List of use cases and associated experiments employed for the evaluation of CohortFinder. This table encompasses three distinct use cases: 1) Tubule segmentation within the NEPTUNE cohort (Pathology), 2) Classification of colon adenocarcinoma in the TCGA-COAD cohort (Pathology), and 3) Segmentation of rectal cancer using cohorts accrued from University Hospitals and Cleveland Clinic (Radiology-MRI).

Metric	Formula
Precision	$\frac{tp}{tp + fp}$
Recall	$\frac{tp}{tp + fn}$
Accuracy	$\frac{tp + tn}{tp + tn + fp + fn}$
IOU (intersection over union)	$\frac{tp}{tp + fn + fp}$
F1 score	$\frac{2 \times Recall \times Precision}{Recall + Precision}$

Supplementary Table 2. Formulas for Quantitative Assessment Metrics. This table provides the mathematical expressions used to compute five key metrics for performance evaluation: Precision, Recall, Accuracy, Intersection Over Union (IOU), and the F1 Score. Definitions included False Positives (FP), False Negatives (FN), True Positives (TP), True Negatives (TN).

			Precision	Recall	Accuracy	IoU	F1 score
Tubule segmentation	Internal testing results	WC	0.93±0.04	0.91±0.14	0.93±0.06	0.85±0.13	**0.91±0.10**
		AC	0.93±0.04	0.94±0.08	0.94±0.03	0.88±0.08	**0.93±0.05**
		BC	0.92±0.05	0.95±0.06	0.94±0.03	0.88±0.07	**0.93±0.05**
	External testing results	WC	0.94±0.02	0.92±0.12	0.93±0.05	0.87±0.11	**0.93±0.09**
		AC	0.94±0.02	0.95±0.06	0.95±0.03	0.89±0.06	0.94±0.04
		BC	0.93±0.02	0.96±0.04	0.95±0.03	0.90±0.05	**0.95±0.03**
Colon adenocarcinoma classification	Internal testing results	WC	0.66±0.38	0.56±0.40	0.56±0.32	0.46±0.36	**0.54±0.38**
		AC	0.89±0.18	0.85±0.23	0.84±0.18	0.77±0.24	0.84±0.20
		BC	0.88±0.18	0.88±0.21	0.85±0.16	0.79±0.23	**0.86±0.19**
	External testing results	WC	0.78±0.26	0.66±0.36	0.66±0.25	0.55±0.32	**0.64±0.32**
		AC	0.90±0.12	0.79±0.25	0.81±0.2	0.73±0.24	0.81±0.21
		BC	0.89±0.13	0.88±0.15	0.86±0.13	0.79±0.16	**0.87±0.11**
Rectal cancer segmentation	Internal testing results	WC	0.69±0.26	0.66±0.28	0.97±0.05	0.48±0.23	0.62±0.24
		AC	0.61±0.29	0.74±0.27	0.95±0.06	0.46±0.23	**0.60±0.24**
		BC	0.67±0.25	0.75±0.26	0.96±0.05	0.51±0.22	**0.64±0.22**
	External testing results	WC	0.62±0.29	0.73±0.30	0.98±0.01	0.50±0.25	**0.62±0.27**
		AC	0.59±0.27	0.83±0.21	0.97±0.05	0.50±0.23	0.63±0.23
		BC	0.63±0.24	0.84±0.18	0.98±0.01	0.55±0.21	**0.68±0.20**

Supplementary Table 3. Summary of performance measures for 3 different use cases, reported on both internal and external testing datasets. For the F1 score, the best performance is highlighted in red, and the worst performance is highlighted in blue.

Tubule segmentation

		Train	Test	Precision	Recall	Accuracy	IoU	F1
Internal testing results	Worst Case	WC_1	WC_2	0.94±0.03	0.92±0.08	0.94±0.04	0.87±0.08	0.93±0.05
			WC_3	0.92±0.05	0.94±0.05	0.94±0.03	0.87±0.07	0.93±0.04
		**WC_2**	WC_1	0.93±0.04	0.82±0.24	0.89±0.10	0.77±0.22	**0.84±0.19**
			WC_3	0.92±0.06	0.94±0.06	0.94±0.03	0.87±0.08	0.93±0.05
		WC_3	WC_1	0.93±0.03	0.87±0.18	0.91±0.07	0.81±0.16	0.88±0.13
			WC_2	0.92±0.03	0.94±0.06	0.94±0.03	0.88±0.07	0.93±0.04
	Average Case	**AC_1**	AC_2	0.93±0.03	0.95±0.05	0.95±0.02	0.88±0.05	**0.94±0.03**
			AC_3	0.92±0.05	0.90±0.14	0.92±0.05	0.83±0.13	0.90±0.10
		AC_2	AC_1	0.93±0.03	0.96±0.03	0.95±0.02	0.90±0.04	0.94±0.03
			AC_3	0.92±0.05	0.93±0.09	0.93±0.04	0.86±0.10	0.92±0.06
		AC_3	AC_1	0.93±0.03	0.96±0.03	0.95±0.02	0.90±0.04	0.94±0.03
			AC_2	0.93±0.03	0.95±0.03	0.95±0.02	0.89±0.04	0.94±0.02
	Best Case	BC_1	BC_2	0.92±0.04	0.93±0.08	0.94±0.03	0.87±0.08	0.93±0.05
			BC_3	0.93±0.03	0.92±0.09	0.93±0.05	0.86±0.09	0.92±0.06
		BC_2	BC_1	0.91±0.06	0.97±0.03	0.95±0.02	0.89±0.06	0.94±0.04
			BC_3	0.92±0.04	0.95±0.06	0.94±0.04	0.88±0.07	0.93±0.04
		BC_3	BC_1	0.91±0.06	0.96±0.03	0.94±0.03	0.88±0.07	0.94±0.04
			BC_2	0.92±0.04	0.95±0.05	0.94±0.02	0.87±0.06	0.93±0.04
External testing dataset	Worst Case	WC_1		0.94±0.02	0.94±0.07	0.94±0.05	0.89±0.07	0.94±0.04
		**WC_2**		0.94±0.02	0.90±0.17	0.93±0.05	0.85±0.16	**0.91±0.13**
		WC_3		0.94±0.02	0.93±0.10	0.93±0.05	0.87±0.09	0.93±0.06
	Average Case	AC_1		0.94±0.03	0.93±0.10	0.94±0.04	0.87±0.09	0.93±0.06
		AC_2		0.93±0.02	0.95±0.05	0.95±0.02	0.89±0.05	0.94±0.03
		AC_3		0.94±0.03	0.95±0.04	0.95±0.03	0.89±0.04	0.94±0.02
	Best Case	BC_1		0.94±0.02	0.94±0.07	0.94±0.04	0.88±0.06	0.94±0.04
		**BC_2**		0.93±0.03	0.97±0.02	0.95±0.02	0.90±0.04	**0.95±0.02**
		BC_3		0.92±0.03	0.96±0.03	0.94±0.02	0.89±0.04	0.94±0.02

Colon adenocarcinoma classification

		Train	Test	Precision	Recall	Accuracy	IoU	F1

Internal testing results	Worst Case	WC_1	WC_2	0.92±0.19	0.85±0.24	0.83±0.22	0.81±0.24	0.87±0.22
			WC_3	0.68±0.33	0.33±0.34	0.57±0.24	0.29±0.29	0.37±0.33
		WC_2	WC_1	0.76±0.27	0.85±0.22	0.69±0.25	0.64±0.28	0.75±0.23
			WC_3	0.53±0.30	0.68±0.36	0.52±0.24	0.43±0.30	0.54±0.31
		**WC_3**	WC_1	0.78±0.32	0.62±0.31	0.65±0.29	0.55±0.30	0.66±0.30
			WC_2	0.28±0.44	0.02±0.07	0.12±0.15	0.02±0.07	**0.03±0.10**
	Average Case	AC_1	AC_2	0.89±0.22	0.79±0.29	0.82±0.20	0.73±0.29	0.80±0.27
			AC_3	0.92±0.15	0.82±0.24	0.83±0.18	0.77±0.24	0.84±0.21
		AC_2	AC_1	0.85±0.17	0.89±0.20	0.85±0.17	0.77±0.22	0.85±0.18
			AC_3	0.89±0.17	0.88±0.18	0.85±0.16	0.80±0.21	0.87±0.17
		AC_3	AC_1	0.86±0.18	0.87±0.22	0.85±0.17	0.78±0.23	0.85±0.20
			AC_2	0.89±0.17	0.85±0.22	0.84±0.17	0.77±0.23	0.84±0.19
	Best Case	**BC_1**	BC_2	0.91±0.17	0.90±0.18	0.89±0.14	0.83±0.21	**0.89±0.18**
			BC_3	0.91±0.14	0.88±0.17	0.86±0.15	0.81±0.20	0.88±0.15
		BC_2	BC_1	0.88±0.17	0.84±0.26	0.82±0.18	0.75±0.26	0.82±0.23
			BC_3	0.90±0.16	0.88±0.18	0.86±0.16	0.80±0.21	0.87±0.16
		BC_3	BC_1	0.82±0.21	0.88±0.23	0.84±0.16	0.76±0.25	0.84±0.21
			BC_2	0.85±0.20	0.88±0.23	0.84±0.19	0.78±0.26	0.85±0.22
External testing dataset	Worst Case	WC_1		0.89±0.13	0.86±0.18	0.84±0.13	0.77±0.19	0.86±0.15
		**WC_2**		0.45±0.38	0.26±0.35	0.31±0.22	0.19±0.25	**0.26±0.31**
		WC_3		0.89±0.13	0.79±0.27	0.79±0.22	0.71±0.26	0.80±0.23
	Average Case	AC_1		0.89±0.14	0.75±0.29	0.78±0.23	0.69±0.28	0.77±0.26
		AC_2		0.90±0.12	0.80±0.23	0.81±0.21	0.73±0.23	0.82±0.19
		AC_3		0.92±0.11	0.82±0.22	0.84±0.17	0.76±0.21	0.84±0.16
	Best Case	**BC_1**		0.91±0.12	0.92±0.09	0.89±0.11	0.85±0.14	**0.91±0.09**
		BC_2		0.90±0.13	0.87±0.19	0.84±0.15	0.78±0.18	0.86±0.13
		BC_3		0.86±0.14	0.85±0.21	0.83±0.15	0.76±0.22	0.84±0.17

Rectal cancer segmentation

		Train	Test	Precision	Recall	Accuracy	IoU	F1
Internal testing results	Worst Case	WC_1	WC_2	0.70±0.24	0.67±0.27	0.97±0.06	0.47±0.21	0.61±0.22
			WC_3	0.69±0.26	0.68±0.30	0.96±0.07	0.50±0.24	0.63±0.26
		WC_2	WC_1	0.74±0.26	0.56±0.27	0.97±0.03	0.45±0.22	0.59±0.24
			WC_3	0.66±0.29	0.60±0.30	0.96±0.04	0.45±0.24	0.58±0.26
		WC_3	WC_1	0.70±0.25	0.69±0.26	0.97±0.02	0.52±0.23	0.65±0.23
			WC_2	0.65±0.27	0.77±0.25	0.97±0.04	0.51±0.22	0.64±0.22
	Average Case	AC_1	AC_2	0.72±0.25	0.68±0.25	0.98±0.02	0.50±0.21	0.64±0.21
			AC_3	0.73±0.25	0.64±0.27	0.96±0.05	0.51±0.22	0.64±0.23
		AC_2	AC_1	0.62±0.27	0.75±0.29	0.96±0.03	0.48±0.23	0.61±0.24
			AC_3	0.70±0.26	0.70±0.30	0.96±0.07	0.50±0.23	0.63±0.23
		**AC_3**	AC_1	0.43±0.26	0.82±0.24	0.92±0.07	0.37±0.22	**0.50±0.25**
			AC_2	0.47±0.27	0.83±0.22	0.94±0.06	0.40±0.23	0.54±0.24
	Best Case	BC_1	BC_2	0.69±0.26	0.72±0.28	0.97±0.06	0.52±0.23	0.64±0.24
			BC_3	0.74±0.23	0.68±0.29	0.96±0.07	0.51±0.23	0.64±0.23
		**BC_2**	BC_1	0.71±0.23	0.73±0.25	0.98±0.02	0.54±0.21	**0.68±0.21**
			BC_3	0.74±0.22	0.68±0.28	0.96±0.07	0.51±0.23	0.64±0.22
		BC_3	BC_1	0.57±0.25	0.86±0.19	0.96±0.02	0.50±0.22	0.63±0.22
			BC_2	0.56±0.27	0.84±0.20	0.96±0.04	0.47±0.22	0.61±0.22
External testing dataset	Worst Case	WC_1		0.66±0.26	0.71±0.28	0.98±0.01	0.52±0.24	0.64±0.24
		**WC_2**		0.58±0.36	0.60±0.37	0.98±0.01	0.43±0.29	**0.54±0.33**
		WC_3		0.62±0.24	0.86±0.18	0.98±0.02	0.55±0.21	0.69±0.20
	Average Case	AC_1		0.66±0.24	0.81±0.16	0.98±0.01	0.55±0.20	0.69±0.19
		AC_2		0.65±0.27	0.79±0.24	0.98±0.01	0.53±0.23	0.66±0.23
		AC_3		0.45±0.26	0.88±0.19	0.94±0.07	0.42±0.23	0.55±0.25
	Best Case	BC_1		0.65±0.25	0.81±0.22	0.98±0.01	0.56±0.22	0.69±0.22
		**BC_2**		0.68±0.22	0.82±0.18	0.98±0.01	0.58±0.19	**0.71±0.17**
		BC_3		0.56±0.24	0.90±0.13	0.97±0.02	0.52±0.21	0.65±0.21

Supplementary Table 4. Detailed performance measures for each experiment for all three use cases. For the F1 score, the best performance is highlighted in red, and the worst performance is highlighted in blue.

Acronym	Full word	Description
CF	CohortFinder	The name of the introduced open-source tool.
BE	Batch effect	Batch effect occurs when non-biological factors in an experiment cause systematic changes in the data produced by the experiment.
WSI	Whole slide images	High-resolution image obtained from scanning a complete histological slide.
DP	Digital pathology	A field of pathology that focuses on data management and analysis of digitalized slide images.
PAS	Periodic acid–Schiff stain	A staining method used in histology to detect polysaccharides such as glycogen in tissues. It results in a magenta color for the structures it stains.
H&E	Hematoxylin and eosin stain	A commonly used stain in histology that shows a broad array of cellular components. Hematoxylin stains nuclei blue, while eosin stains the cytoplasm pink.
MRI	Magnetic Resonance Imaging	A noninvasive medical imaging test that produces detailed images of almost every internal structure in the human body, including the organs, bones, muscles, and blood vessels.
CT	Computed Tomography	An imaging method that uses x-rays to create detailed pictures of cross-sections of the body, useful in diagnosing diseases and conditions such as cancers.
PET	Positron Emission Tomography	A type of nuclear medicine imaging that measures metabolic activity in tissues, often used in detecting cancer, brain disorders, and heart conditions.
ML	Machine learning	The study and construction of algorithms that can learn from and make predictions on data.
DL	Deep learning	A subset of machine learning that uses neural networks with many layers to learn from data.
BC	Best case	The data partitioning scenario where batch effects are most optimally mitigated.
AC	Average case	The data partitioning scenario generated by random assignment.
WC	Worst case	The worst data partitioning scenario where the data of training/testing cohort are mutually and exclusively from the same BE group.
TN	True negative	This occurs when the model correctly predicts the absence of a feature or condition, such as correctly identifying that a segment of an image does not contain pathological tissue or accurately classifying an image as healthy.
TP	True positive	This is when the model correctly identifies a feature or condition present in the medical image, such as accurately recognizing a tumor in a segmentation task or correctly classifying an image as indicative of disease.
FN	False negative	This is when the model fails to identify a feature or condition that is actually present, such as not detecting a tumor that exists in a segmentation task or failing to classify a diseased image correctly.
FP	False positive	This happens when the model incorrectly identifies a feature or condition as present, such as mistakenly delineating a region as a tumor in a healthy tissue segment or classifying a healthy image as showing signs of disease.

Supplementary Table 5. Acronyms and definitions utilized in the manuscript.

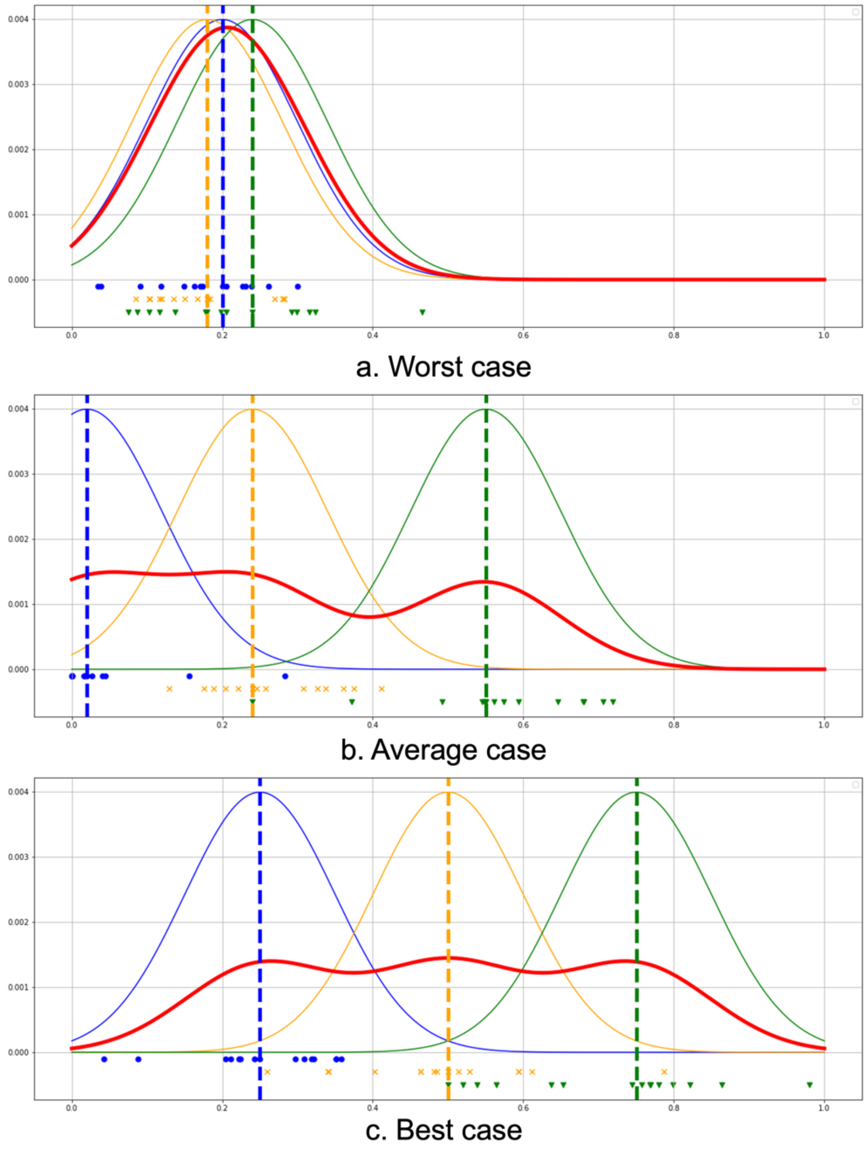

Supplementary Figure 1 Simulated example to demonstrate the combined distribution-level effects of data augmentation for three sampling techniques: (a) worst-case, (b) average-case, and (c) best-case (CohortFinder). The x-axis represents the value of a quantitative measurement (e.g., brightness) having a domain of [0,1]. The star markers at the top of the plot represent the values of that metric for 9 available data samples, from which each sampling technique selects a subset of 3 (here the diamond-shaped markers (blue, orange, and green)). The remaining smaller markers (circles, squares, and triangles) represent instantiations of Gaussian data-augmented versions of each of the 3 selected markers, with their associated mean identified by a dashed vertical line. The y-axis represents the density of these markers both at a slide level (orange, blue, green curves), and the cumulative density distribution (red curve, sum of the other 3 curves). It can be noted that in the (a) worst case, due to the over-representation of data points towards the left of the plot, the red curve does not cover the entire domain, intuitively implying that an ML model would not be exposed to measurement values greater than 0.5. In the (b) average case, though the red curve expands towards the right, the system still lacks instances with measurement values greater than 0.80. Notably, because this average case arises from random sampling, it carries an inherent risk of being the worst-case scenario as well. Conversely, employing CohortFinder leads to (c) the best case, which intentionally selects representative samples such that the coverage of the domain is more complete, potentially improving the generalizability of an ML model trained from such data. This is to say that intuitively, CohortFinder helps identify the “best" samples from which to perform data augmentation, thus yielding greater coverage of the sample space.

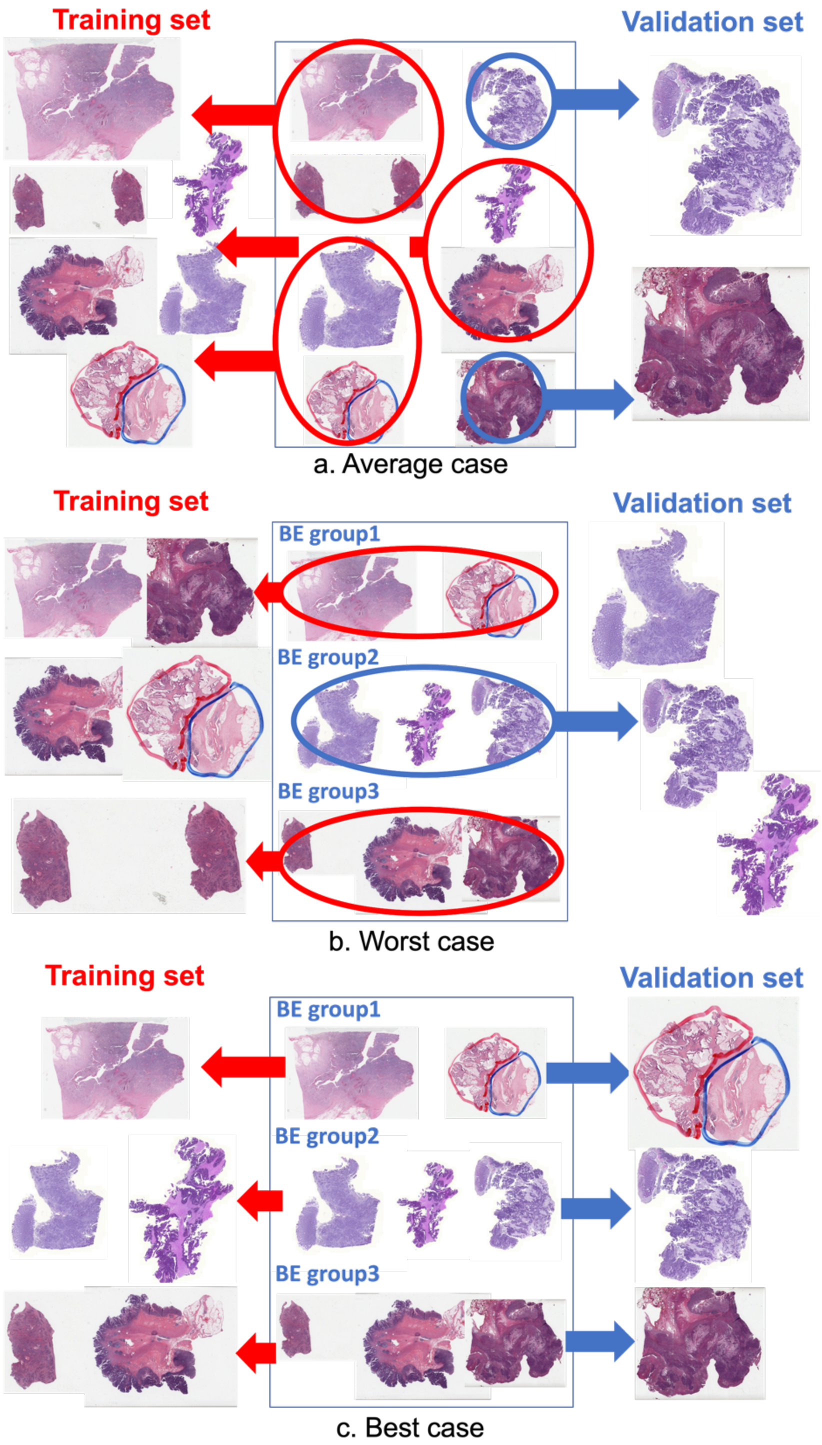

Supplementary Figure 2 *Three data partitioning strategies. (a) Average case: Patients are randomly split into training and testing sets without considering BEs, which can cause possible sub-optimal situations. (b) Worst case: patients with similar BE metrics are exclusively assigned to the training or testing dataset, resulting in slides in the training set looking highly dissimilar to those in the testing set. (c) Best case: where detected BE-groups are systematically divided between training/testing sets. This process, enabled via CF, ensures the diversity of the training dataset, and thus, improves the robustness of the machine model.*

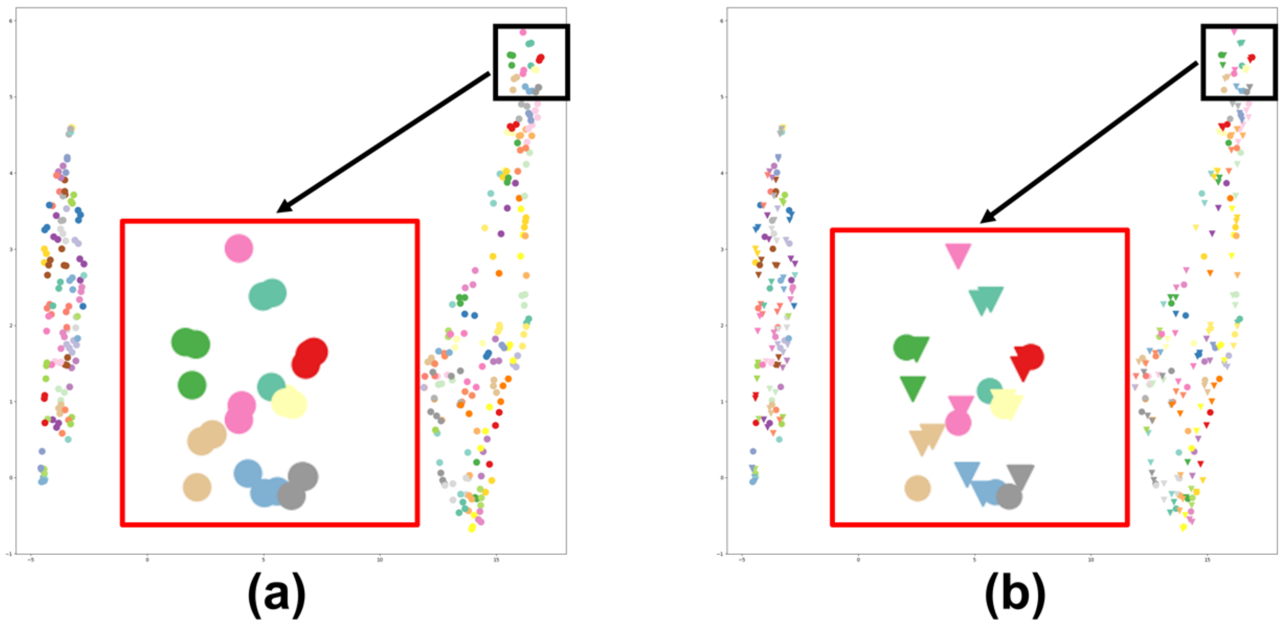

Supplementary Figure 3. Detail of UMAP plots generated via CohortFinder for the colon adenocarcinoma classification use case (a) Quality measures embedded in a 2-dimensional plot using U-MAP. (b) Dots replaced with "v (triangle-down)" and "o (circle)", where "v" indicates a slide to be placed into the training set and "o" indicates a slide to be placed in the testing set.

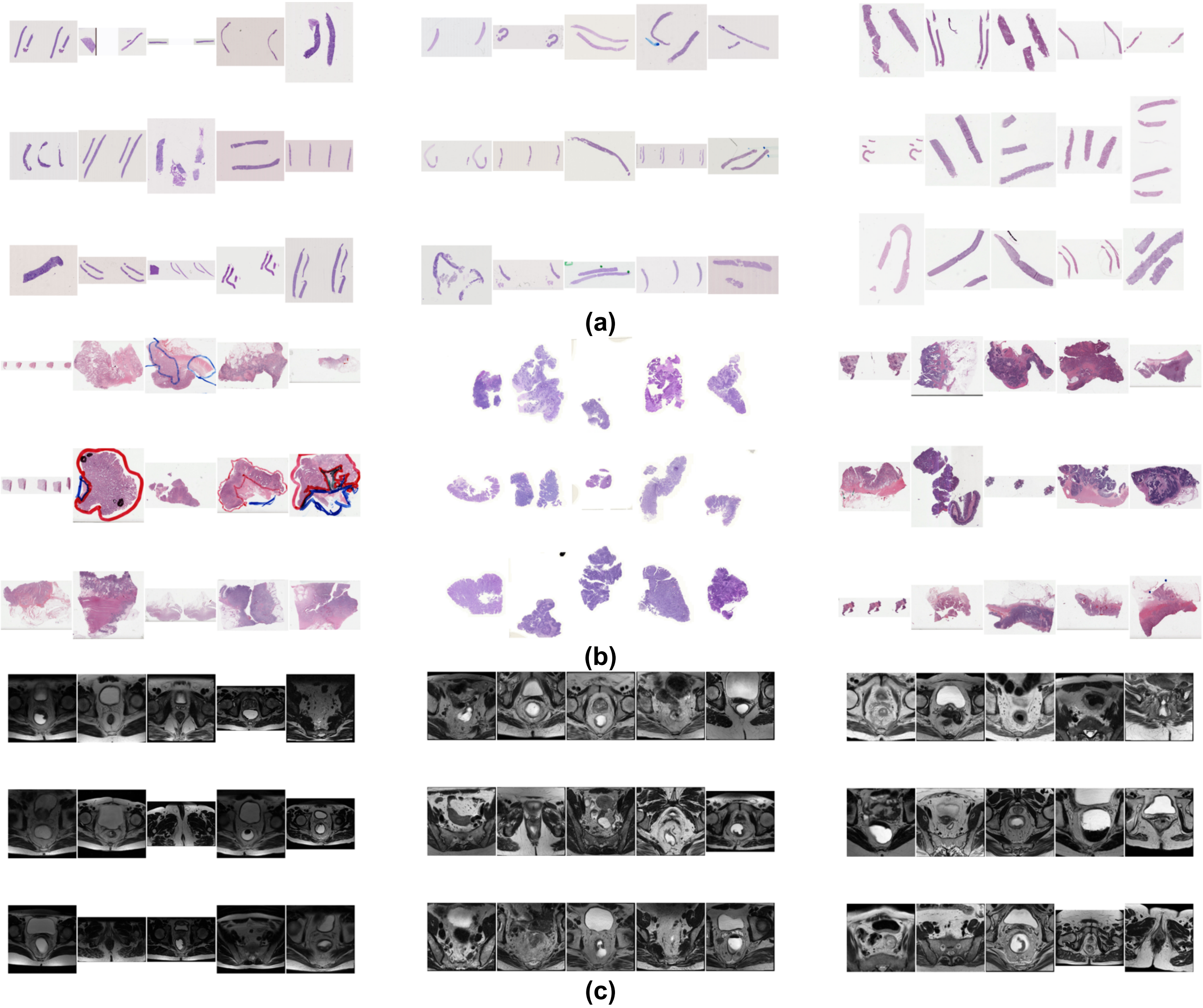

Supplementary Figure 4. Three contact sheets produced by CohortFinder for (a) Tubule segmentation on WSIs (b) Colon adenocarcinoma classification on WSIs (c) Rectal cancer segmentation on MRIs. Each row shows 3 detected BE-groups with notable (i) intra-group homogeneity and (ii) inter-group heterogeneity, providing a visual confirmation of successful BE-group detection.

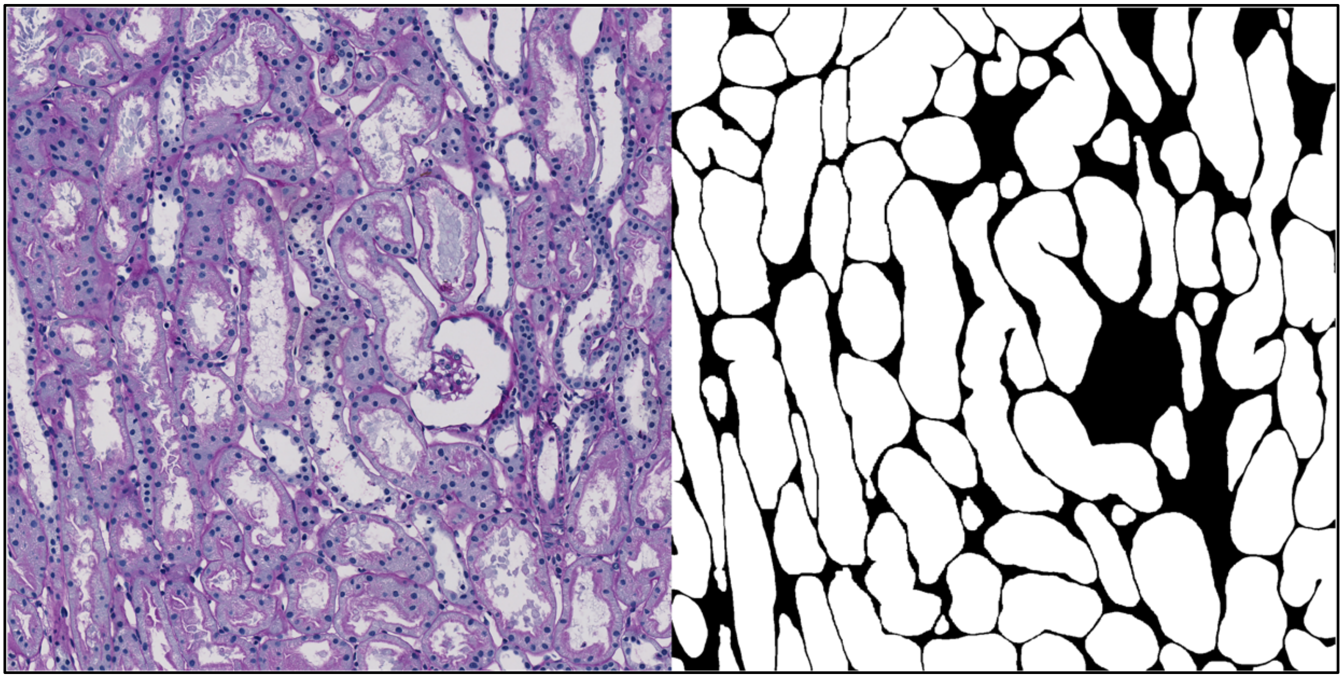

Supplementary Figure 5. Ground truth example for the tubule segmentation use case, the left one is the cropped ROI, and the right one is the expert annotation for the renal tubules.

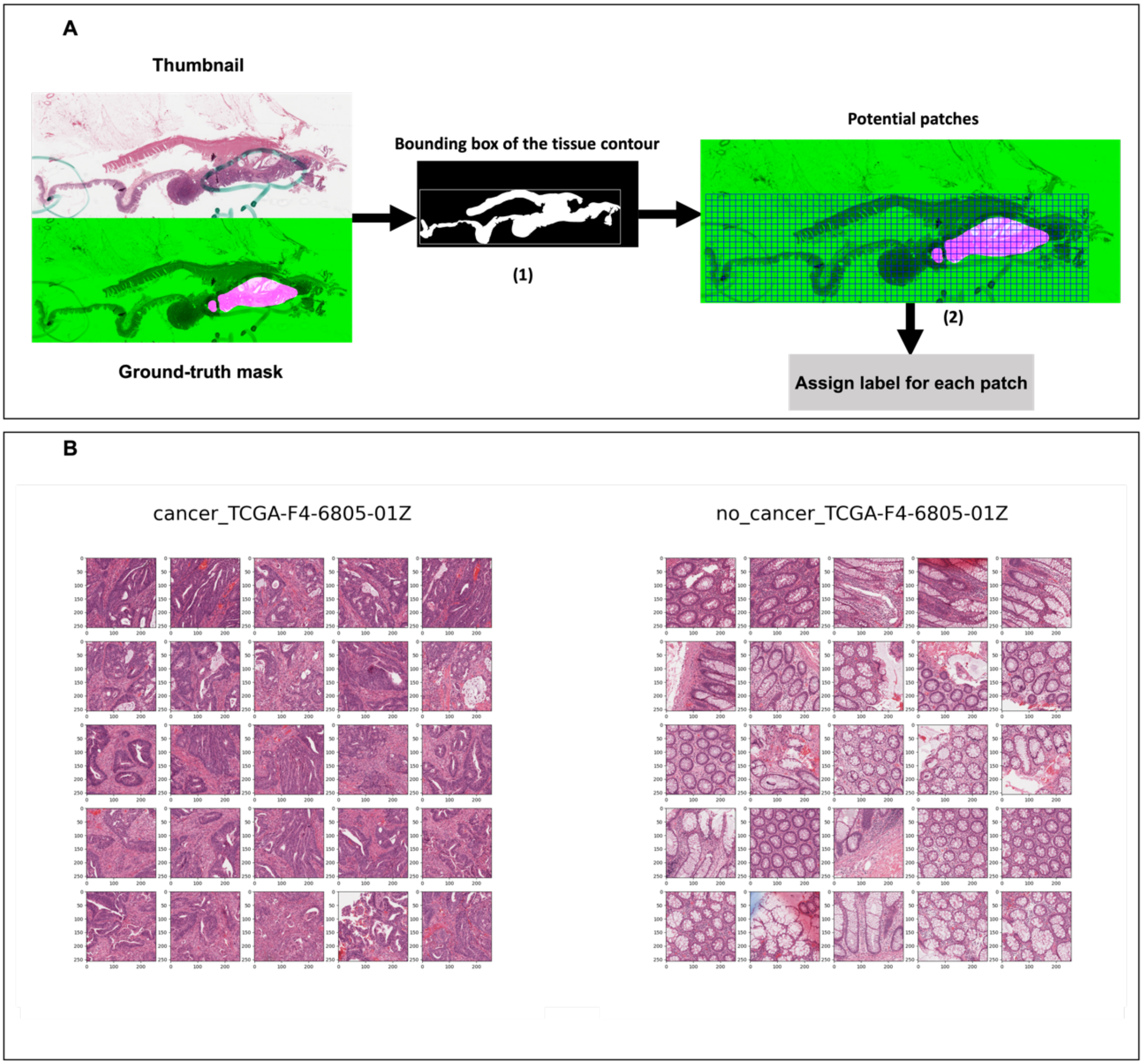

Supplementary Figure 6. Overview of patch extraction for colon cancer classification. Panel A demonstrates the patch extraction workflow: the upper left image represents a downscaled thumbnail from the whole slide image (WSI), and below it, the pathologist's annotated ground truth mask with cancerous regions marked in fuchsia. The central image depicts the tissue mask as identified by HistoQC alongside the delineated bounding box encompassing the tissue region. The image on the right displays all possible patches derived from the thumbnail within the bounding box through tessellation. Each patch is categorized as positive or negative based on the percentage of cancerous tissue (the threshold is set as 90%). The patch is labeled as non-informative and excluded from the training/testing cohort if: a) <10% area is intersected with the detected tissue mask, and b) the maximum difference of color density for all the 3 color channels is less than 20. Panel B shows selected patch samples illustrating the distinction between cancerous and non-cancerous tissues from the patient identified by the code TCGA-F4-6805-01Z.

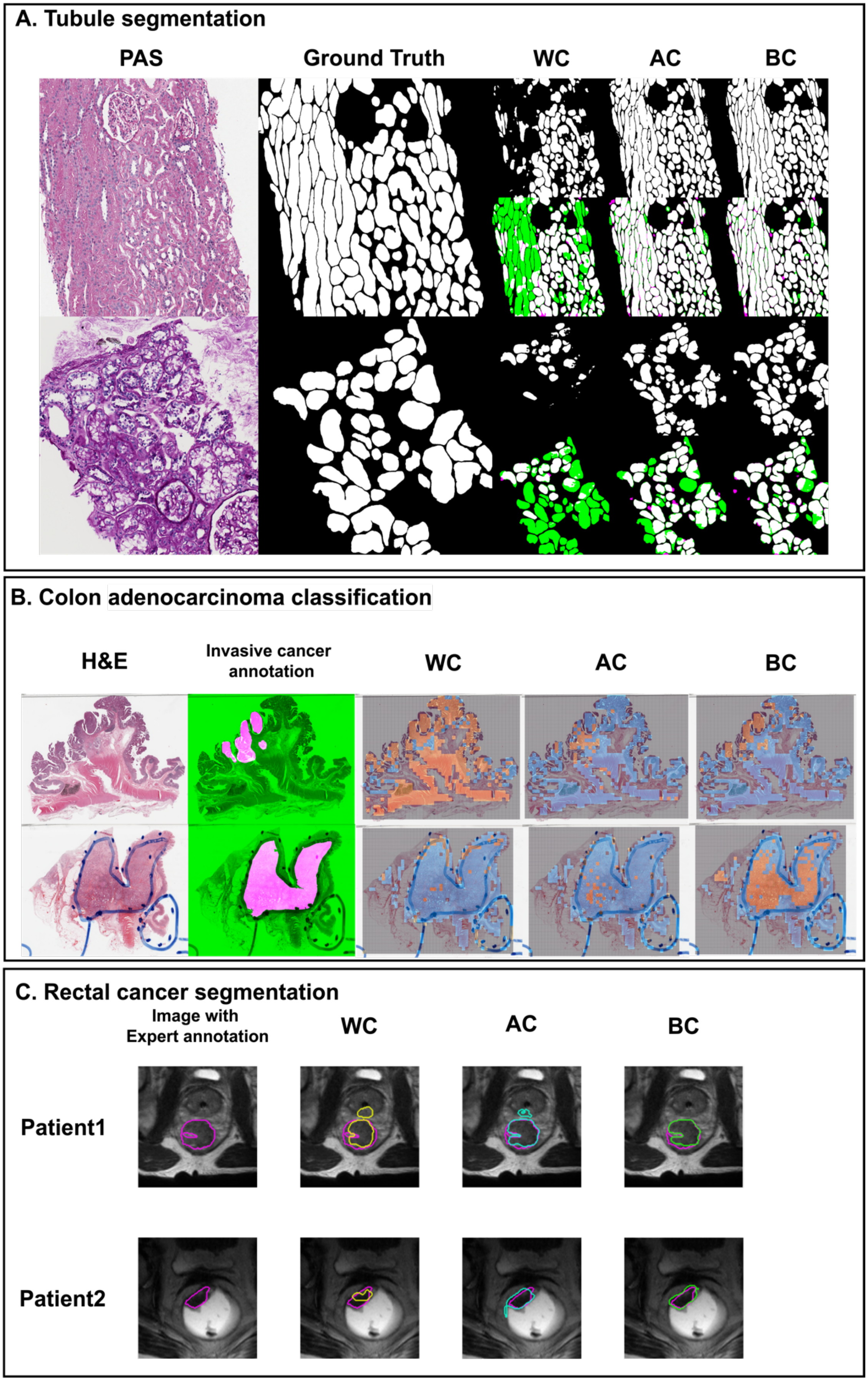

Supplementary Figure 7. *Qualitative comparison of the three use cases in the external set. A) For tubule segmentation task. The first column is a 3000x3000 PAS-stained ROI cropped at 40x, the second column is the tubule segmentation ground truth (GT). The remaining images are the results of BC, AC, and WC. In each scenario, the top row is the DL model results, while the bottom row corresponds to the overlay image of DL output images with the GT, where green represents the false*

negative (FN) area, and the fuchsia represents the false positive (FP) area. B) For the colon cancer classification task. The first column represents the thumbnails of the H&E-stained WSI, and the second column is the cancer region annotation overlaid in fuchsia. The remaining images are the heatmap results of different BC, AC, and WC, where the detected cancer area is highlighted as orange, the non-cancer area is highlighted as blue, while the gray area corresponds to non-informative (representing background or non-tissue). C) For the rectal tumor segmentation task. The first column corresponds to the patient's MRI with the expert tumor annotation contoured in fuchsia. The remaining images are rectal tumor segmentations via WC (yellow), AC (cyan), and BC (green) models compared to expert annotations (fuchsia).

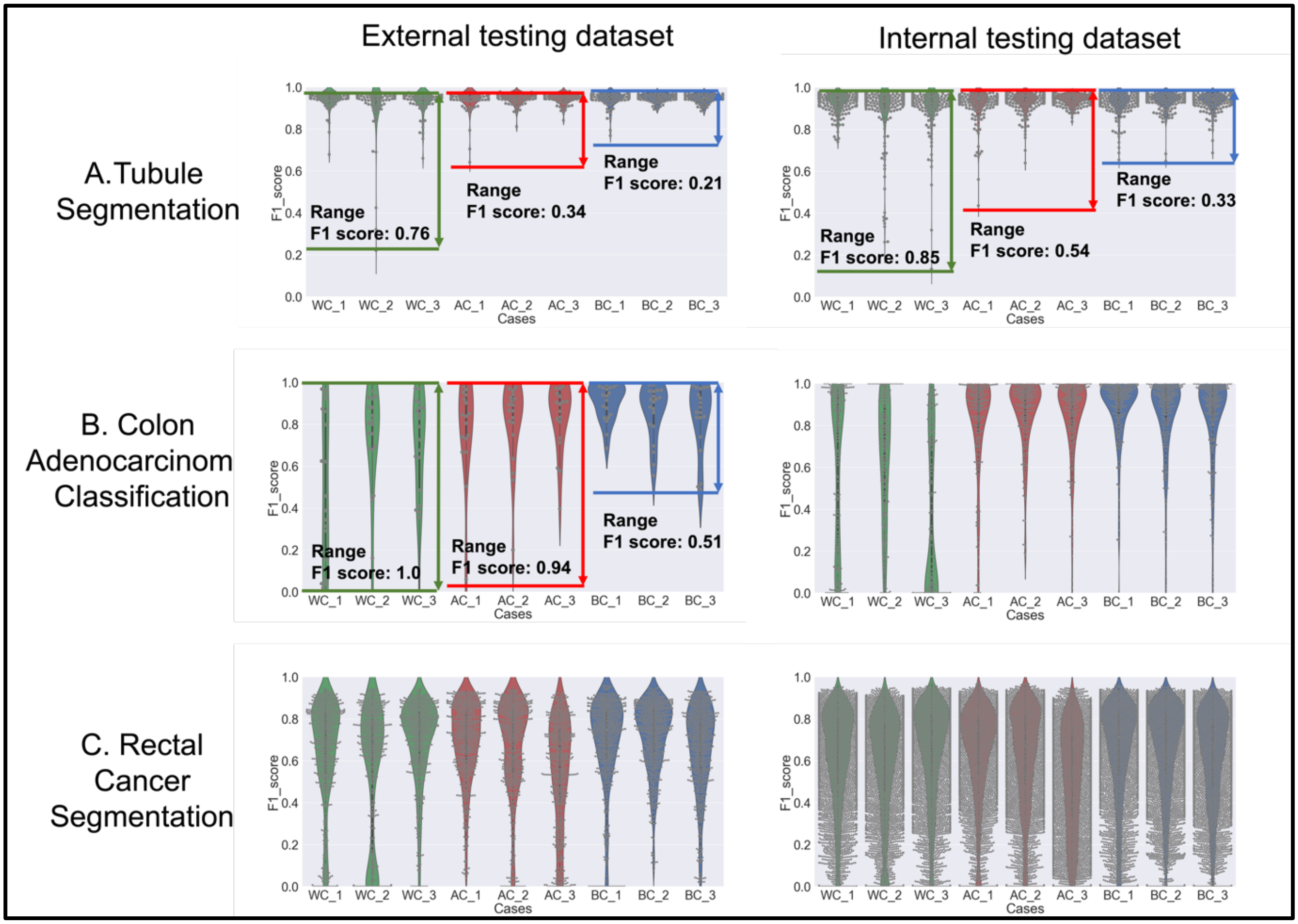

Supplementary Figure 8. Violin plots of the F1-score evaluation measure distribution for both external and internal testing datasets and for each use case. Each dot represents the F1-score value for (a) tubule segmentation at the ROI level, (b) colon adenocarcinoma classification at the WSI level and (c) rectal cancer segmentation at slice level. In a majority of the comparisons, the BC distribution appears more compact than WC and AC distributions.

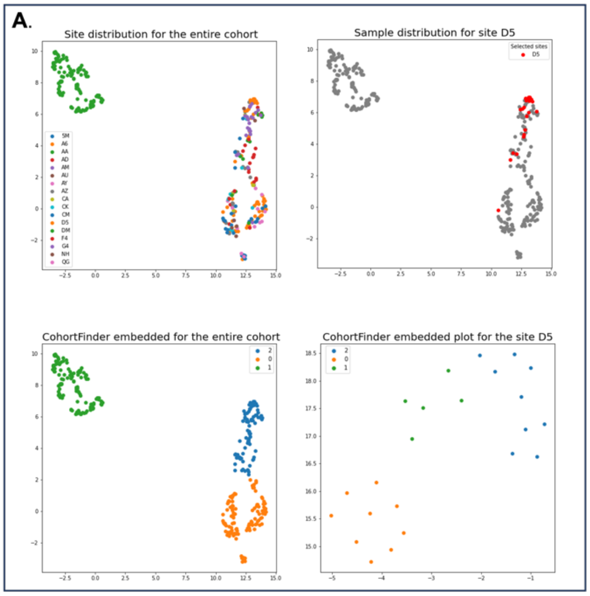

B.

	Silhouette Coefficient	Davies-Bouldin Index	Calinski-Harabasz Index
Entire_cohort_BE_group=3	**0.70**	**0.44**	**3444.73**
SITE_D5_BE_group=3	0.52	0.64	58.47

C.

Measurement name	Description	Indication of BEs
Silhouette Coefficient	Measures how similar an object is to its own cluster as compared to other clusters. Values range from -1 to 1.	Values close to 1 indicate a worse BEs.
Davies-Bouldin Index	Measures how similar an object is to its own cluster compared to other clusters. Values ranges from -1 to 1.	Lower values indicate worse BEs.
Calinski-Harabasz Index	Assesses the ratio of between-cluster variance to within-cluster variance. Values range from 0 to infinity.	Higher values indicate worse BEs.

Supplementary Figure 9 presents preliminary results for quantification of BE severity via clustering metrics, using the TCGA-COAD cohort as an example. Panel A (from left to right), the first row sequentially displays CohortFinder embedding results for the site distribution for the entire cohort with different colors highlighting different sites, and the sample distribution for site D5 alone (highlighted in red). CohortFinder was then run with the same parameter settings individually on (a) the entire cohort, and (b) only samples from site D5. The second row displays the associated CohortFinder embedding plots for the entire cohort and D5, separately, where the green, blue, orange represent different BE groups generated by CohortFinder. In panel B, three clustering evaluation metrics are presented in a table for the 2 scenarios. Within this table, the best score for each metric is highlighted in red. The entire cohort scenario has higher BE scores for all three clustering metrics, indicating more significant batch effects being present compared to an individual site (D5). Panel C shows a table with the names of the measurements being utilized for BE scoring, their descriptions, and the values indicative of good clustering for each of the three measurements: Silhouette coefficient, Davies-Bouldin index, and Calinski-Harabasz index.

References

1. Chen Y, Zee J, Smith A, et al. Assessment of a computerized quantitative quality control tool for whole slide images of kidney biopsies. *J Pathol*. 2021;253(3):268-278. doi:10.1002/path.5590

2. Barisoni L, Gimpel C, Kain R, et al. Digital pathology imaging as a novel platform for standardization and globalization of quantitative nephropathology. *Clin Kidney J*. 2017;10(2):176-187. doi:10.1093/ckj/sfw129

3. Barisoni L, Nast CC, Jennette JC, et al. Digital pathology evaluation in the multicenter Nephrotic Syndrome Study Network (NEPTUNE). *Clin J Am Soc Nephrol*. 2013;8(8):1449-1459. doi:10.2215/CJN.08370812

4. Gadegbeku CA, Gipson DS, Holzman LB, et al. Design of the Nephrotic Syndrome Study Network (NEPTUNE) to evaluate primary glomerular nephropathy by a multidisciplinary approach. *Kidney Int*. 2013;83(4):749-756. doi:10.1038/ki.2012.428

5. Jayapandian CP, Chen Y, Janowczyk AR, et al. Development and evaluation of deep learning–based segmentation of histologic structures in the kidney cortex with multiple histologic stains. *Kidney International*. 2021;99(1):86-101. doi:10.1016/j.kint.2020.07.044

6. Sirinukunwattana K, Domingo E, Richman SD, et al. Image-based consensus molecular subtype (imCMS) classification of colorectal cancer using deep learning. Gut. 2021;70(3):544-554. doi:10.1136/gutjnl-2019-319866

7. Janowczyk A, Zuo R, Gilmore H, Feldman M, Madabhushi A. HistoQC: An Open-Source Quality Control Tool for Digital Pathology Slides. *JCO Clin Cancer Inform*. 2019;3:CCI.18.00157. doi:10.1200/CCI.18.00157

8. Fedorov A, Beichel R, Kalpathy-Cramer J, et al. 3D Slicer as an image computing platform for the Quantitative Imaging Network. *Magn Reson Imaging*. 2012;30(9):1323-1341. doi:10.1016/j.mri.2012.05.001

9. Firman Ashari, Ilham & Nugroho, Eko & Baraku, Randi & Yanda, Ilham & Liwardana, Ridho. (2023). Analysis of Elbow, Silhouette, Davies-Bouldin, Calinski-Harabasz, and Rand-Index Evaluation on K-Means Algorithm for Classifying Flood-Affected Areas in Jakarta. Journal of Applied Informatics and Computing. 7. 89-97. 10.30871/jaic.v7i1.4947.
